# A Flexible Approach for the Analysis of Rare Variants Allowing for a Mixture of Effects on Binary or Quantitative Traits

**DOI:** 10.1371/journal.pgen.1003694

**Published:** 2013-08-15

**Authors:** Geraldine M. Clarke, Manuel A. Rivas, Andrew P. Morris

**Affiliations:** Wellcome Trust Centre for Human Genetics, University of Oxford, Oxford, United Kingdom; Wellcome Trust Sanger Institute, United Kingdom

## Abstract

Multiple rare variants either within or across genes have been hypothesised to collectively influence complex human traits. The increasing availability of high throughput sequencing technologies offers the opportunity to study the effect of rare variants on these traits. However, appropriate and computationally efficient analytical methods are required to account for collections of rare variants that display a combination of protective, deleterious and null effects on the trait. We have developed a novel method for the analysis of rare genetic variation in a gene, region or pathway that, by simply aggregating summary statistics at each variant, can: (i) test for the presence of a mixture of effects on a trait; (ii) be applied to both binary and quantitative traits in population-based and family-based data; (iii) adjust for covariates to allow for non-genetic risk factors and; (iv) incorporate imputed genetic variation. In addition, for preliminary identification of promising genes, the method can be applied to association summary statistics, available from meta-analysis of published data, for example, without the need for individual level genotype data. Through simulation, we show that our method is immune to the presence of bi-directional effects, with no apparent loss in power across a range of different mixtures, and can achieve greater power than existing approaches as long as summary statistics at each variant are robust. We apply our method to investigate association of type-1 diabetes with imputed rare variants within genes in the major histocompatibility complex using genotype data from the Wellcome Trust Case Control Consortium.

## Author Summary

Rapid advances in sequencing technology mean that it is now possible to directly assay rare genetic variation. In addition, the availability of almost fully sequenced human genomes by the 1000 Genomes Project allows genotyping at rare variants that are not present on arrays commonly used in genome-wide association studies. Rare variants within a gene or region may act to collectively influence a complex trait. Methods for testing these rare variants should be able to account for a combination of those that serve to either increase, decrease or have no effect on the trait of interest. Here, we introduce a method for the analysis of a collection of rare genetic variants, within a gene or region, which assesses evidence for a mixture of effects. Our method simply aggregates summary statistics at each variant and, as such, can be applied to both population and family-based data, to binary or quantitative traits and to either directly genotyped or imputed data. In addition, it does not require individual level genotype or phenotype data, and can be adjusted for non-genetic risk factors. We illustrate our approach by examining imputed rare variants in the major histocompatibility complex for association with type-1 diabetes using genotype data from the Wellcome Trust case Control Consortium.

## Introduction

Despite the recent successes of genome-wide association studies (GWAS), which can be well powered under the common disease, common variant hypothesis, the majority of the genetic component of many complex traits remains unexplained. For example, hundreds of common genetic variants, in at least 180 loci, have been associated with height in studies of up to more than 180,000 individuals. However, the individual effects of these variants are modest and their cumulative effect explains just over 10% of the phenotypic variation in height [1], [2], [3], [4]. Rare variants may play an important role in explaining the “missing heritability” of complex traits. Due to recent advances in high-throughput re-sequencing technology, it is becoming financially feasible to assay rare genetic variation in thousands of individuals on the scale of the whole-exome, or even the whole genome. Furthermore, with the availability of whole-genome re-sequencing reference panels, such as those made available through the 1000 Genomes Project [5], imputation allows the possibility to predict genotypes at rare variants not present on, or captured by, GWAS genotyping arrays. Therefore, we now have an exciting opportunity to explore a range of models that may help to explain the missing heritability of complex traits using rare genetic variation. One such model is that where a gene or region affects a complex trait as a consequence of the combined effects of its constituent rare variants. The effects at each rare variant can be either modest or highly penetrant, and can act to either increase or decrease the trait or disease risk.

Recently published methods for the analysis of multiple rare variants illustrate that power can be greatly increased by combining information in a joint analysis in comparison to studying individual variants one at a time [6], [7], [8], [9], [10], [11]. These so called “burden tests” are optimal when all variants have the same direction of effect. However, these variants may act individually to either increase or decrease trait values, or they may be neutral (i.e. no effect on the trait). Ideally, we wish to test for the presence of a mixture of increaser, decreaser and neutral effects at multiple rare variants on a complex binary or quantitative trait. Zelterman and Chen [12] describe tests of homogeneity against such central mixture alternatives for general sampling distributions that are based on the score function. These so called “C-alpha” tests are powerful for detecting the presence of central mixtures [13]. Neale et al. [14] proposed a C-alpha test for the analysis of sequence level data for association with binary (disease) traits based on binomially distributed measures of effect at each site. Their approach has the advantage of allowing for a mixture of risk, protective and neutral effects, but cannot explicitly be applied to quantitative traits, account for non-genetic risk factors as covariates, or allow for imputed variation. More recently, score-based variance component tests SKAT (sequence kernel association test) [15] and an optimized version (SKAT-O) [16] have been proposed for the detection of a mixture of effects which can be applied to both binary and quantitative traits and which can adjust for covariates. These tests have been shown to outperform burden tests and the Binomial C-alpha test in a wide range of scenarios.

Here, we introduce a C-alpha test for the analysis of rare genetic variation for association with both binary and quantitative traits based on normally distributed measures of effect at each site. Measures of effect at each site can be calculated from re-sequencing, array genotyping or imputed data or taken directly from summary measures of effect available, for example, from meta-analysis or published data. Our test assesses the evidence for a mixture of increaser, decreaser and neutral effects in a gene, region or pathway and can be applied to both population and family-based association studies and can adjust for covariates to allow for non-genetic risk factors, such as indicators of population stratification. We refer to our test as the *Generalised C-alpha* test. We report the results of simulations to investigate the power of our test to detect rare variant association with a quantitative trait, and compare performance with existing approaches.

The HLA class II genes in the major histocompatibility locus (MHC) play a major role in susceptibility to type-1 diabetes (T1D) [17], but common variants mapping to other genes in this region have also been implicated in the disease. Imputation into existing GWAS genotype data up to publicly available reference panels of sequence data can be used to identify novel and refined signals of association with common SNPs (MAF>1%) [18] and is feasible for the evaluation of rare variants [19]. We have used our Generalised C-alpha test to evaluate the evidence for rare variant association with T1D within genes in the MHC using GWAS genotype data from the Wellcome Trust Control Consortium (WTCCC) [20] imputed up to reference panels made available through the 1000 Genomes Project [5].

## Materials and Methods

### Generalised C-alpha Test

Consider a gene, region or pathway containing *K* variants, each with a minor allele frequency (MAF) less than a pre-defined threshold and assayed in a sample of individuals measured for a binary or a quantitative trait. Suppose that at each variant a normally distributed estimate of the effect of the minor allele on the trait of interest can be obtained. For example, in a case-control association study such an estimate may be the log allelic odds ratio obtained as a coefficient in a logistic regression; or in a quantitative trait association study, the estimate may be the per-allele increase in phenotypic value obtained as a coefficient in a linear regression. For each variant alone, there is unlikely to be enough information to make inference about association, unless the sample size is unfeasibly large. However, if the gene is not associated with the trait, then the distribution of estimates across all variants will be Gaussian with mean zero. Conversely, if variants in the gene are associated with the trait, there will be a mixture of Gaussian distributions with different means, manifested as “overdispersion”, which can be detected by a C-alpha test.

More formally, let 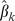 denote the effect estimate, and 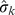 it's corresponding estimate of standard deviation, at variant *k*, *k* = 1,…,*K*. We assume that 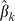 are independent Gaussian distributed random variables with mean 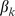 and standard deviation 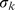. As described, such estimates will typically have been obtained from a logistic (binary trait) or linear (quantitative trait) regression of trait value on genotype. The C-alpha test of homogeneity can be derived for a given sampling model. Here the effects are treated as sampling units from a Gaussian sampling model. Under the null hypothesis of no association with the trait, we assume that all 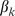 are equal to some fixed, unknown value, denoted 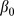. Under the alternative hypothesis, we assume that the 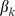 take on a mixture of values, centred at 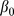. The C-alpha test statistic for a test of homogeneity of 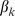 against a central mixture of alternative Gaussian hypotheses is
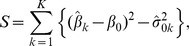
where 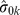 is an estimate of 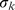 under the null hypothesis. In practice, we estimate 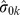 by the observed standard deviation 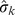. Notice that *S* is simply the sum of the differences between the variance of the observed measures of association and the expected variance under the null hypothesis. To standardise *S*, we require the estimated normalizing variance
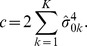
The standardised C-alpha test statistic is then
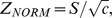
which is asymptotically standard Gaussian distributed. The null hypothesis of no association is rejected for values of Z_NORM_ significantly larger than that expected using a one-tailed test of size *α*. The quantities *S* and *c* are easily derived using methods detailed in Zelterman and Chen [12] for sampling units from a distribution belonging to the exponential family: in this case, the Gaussian distribution, 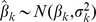 where 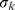 is treated as a nuisance parameter. Note that a natural adjustment for the effect of non-genetic risk factors can be achieved by including covariates in the regression model used to estimate 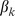. Furthermore, we can consider imputed variation by replacing direct genotypes with dosages under an additive model, or by maximisation of the missing data likelihood of the distribution of genotypes.

For genetic association studies, the expected effect of a minor allele is zero, so that 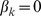, and the C-alpha statistic reduces to:
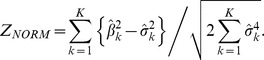
The assumption that the distribution of Z_NORM_ is Gaussian depends on: (i) the degree of sparseness in the data, as summarised by the relationship between sample size and MAF at each variant; (ii) the number of variants that are considered and (iii) the independence of variants. When the data are too sparse, because the sample size is too small and/or the MAF too low, the maximum likelihood estimates of effect size computed at each site are typically unstable. Furthermore, the discrepancy between the empirical variance of the estimates, and their variance under the reference asymptotic distribution can be large, resulting in inaccurate type I error [21]. It is reasonable to assume that large numbers of individuals will be genotyped because in a practical study design, tests require large numbers of individuals for adequate power, however the minimum MAF must be constrained to ensure stability of estimates in the presence of, for example, private mutations. The second and third requirements ensure convergence of the null distribution of the Z_NORM_ to Gaussian by the central limit theorem. To estimate significance accurately for low MAF, where small numbers of variants are considered or where variants are correlated, standard permutation testing is required. See Text S1 for details of the standard permutation approach utilised here.

### Simulation Study

We conduct simulations to investigate the performance of the Generalised C-alpha test for the identification of rare variants associated with a binary or quantitative trait. We compare the performance of the Generalised C-alpha test to three existing approaches: (i) the optimized score-based variance component test (SKAT-O, by Lee et al. [15] (ii) the Binomial C-alpha rare variant test by Neale et al. [14], and (iii) GRANVIL, a burden test of association of binary or quantitative traits with accumulations of minor alleles at rare variants in a generalised linear modelling framework by Morris and Zeggini [10]. A short summary of these tests is given here.

- *SKAT-O* performs a test of association between genetic variants in a region and binary or continuous traits using kernel machine methods. SKAT-O aggregates individual score test statistics obtained at each variant to compute an overall p-value for the region. SKAT-O can be applied to imputed data and can allow adjustment for covariates.
- The *Binomial C-alpha* test is a rare variant test developed for binary (disease) traits. The test models the number of minor alleles, *y_k_*, at variant *k* out of a total of *n_k_* observations by a binomial (*n_k_*, *p_k_*) distribution, where *k* = 1,…,*K*. Under the null hypothesis, *p_k_* = *p_0_*, the proportion of cases present in the sample. Under the alternative hypothesis, *p_k_* can take on a mixture of values across the *K* variants, with some variants deleterious (i.e. with greater frequency in the cases than controls, *p_k_*>*p_0_*), some protective (i.e. with greater frequency in the controls than the cases *p_k_*<*p_0_*), and some neutral (i.e. with equal frequency in cases and controls *p_k_* = *p_0_*). It can then be shown that the Binomial C-alpha test statistic is simply:
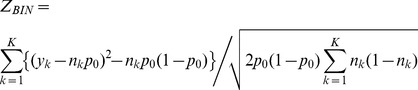
Z*_BIN_* has a standard Gaussian distribution under the null hypothesis of no association, which is rejected for values of Z*_BIN_* significantly larger than that expected for a one-tailed test of size *α*. The Binomial C-alpha test cannot adjust for covariates and cannot be directly applied to imputed data.
- *GRANVIL* models the trait value of an individual as a function of the proportion of rare variants at which they carry at least one minor allele in a generalised linear regression framework. GRANVIL can thus be applied to binary and quantitative traits, can incorporate imputed genotypes, and can allow adjustment for covariates. However, GRANVIL is a burden test, and thus assumes the direction of effect of all rare variants is the same, within the same gene or pathway.

Our simulations make use of a simple model of population genetics to generate high-density haplotype data in 30–200 kb genomic regions, designed to represent a gene. Haplotypes are then randomly paired together to form individuals for analysis, and quantitative trait values are generated according to their genotypes at rare causal variants, selected at random according to the underlying trait association model. In the trait association model that we consider here, we assume that the expected phenotypic value of an individual is determined by the net effect of a combination of increaser causal variants, which serve to elevate the mean trait value in the population, and decreaser causal variants, which serve to reduce it. The trait association model is parameterised in terms of: (i) the maximum MAF of each individual causal variant; (ii) the total MAF of all causal variants in the gene; (iii) the relative proportion of increaser and decreaser causal variants; and (iv) the joint contribution of the causal variants in the gene to the trait variance. Full details of the simulation process are described in Text S1.

The Generalised C-alpha test, SKAT-O and GRANVIL are applied directly to the simulated quantitative trait. However, to apply tests designed for binary traits, we dichotomise the quantitative distribution by assigning individuals as “cases” if they belong to the upper 50% of the trait distribution, or “controls” otherwise. The Generalised C-alpha test, as well as the Binomial C-alpha test, is then applied to the dichotomised trait. The significance of the Generalised C-alpha and Binomial C-alpha test statistics are evaluated empirically by standard permutation testing (see Text S1 for details), whilst GRANVIL relies on the asymptotic properties of a linear regression model and SKAT-O uses Davies method [22] for approximating the distribution of the test statistic. For each simulation, we permute 1,000 or 100,000 times to ensure accurate assessment at 0.05 and 1×10^−5^ significance levels, respectively. Simulations are repeated 10,000 times for each set of parameter values.

### Rare Variant Analysis of Imputed Data with T1D

We evaluated the evidence for rare variant (MAF<1%) signals of association with T1D in genes on chromosome 6 using the Generalised C-alpha test applied to rare variants using genotype data from the WTCCC [18]. All WTCCC samples are ascertained from the UK. We applied the same quality control (QC) filters employed and described by the WTCCC to exclude samples and SNPs from the analysis. These high-quality samples were imputed up to the Phase 1 1000 Genomes Project reference panel (June 2011 interim release) [5] comprising 1,094 phased individuals from multiple ancestry groups. Adjustment for fine-scale population structure is critical in rare variant analysis because recent founder effects can exert greater impact on association analyses with rare variants than with common variants [23]. To control for population structure we constructed principal components to represent axes of genetic variation within the UK and included these as covariates in association analyses to obtain estimates of effect at each SNP that are adjusted for ancestry. These procedures for imputation and control of fine-scale population structure are the same as those utilised by Magi et al. [24], full details of which are presented in their paper.

For each gene, the Generalised C-alpha test was applied to SNPs in two MAF ranges: 0.1%<MAF<0.5% (very rare) and 0.5%<MAF<1% (rare). Measures of effect at each SNP used in the Generalised C-alpha test were the log odds ratios estimated from single SNP additive tests of association using simple logistic regression. The Generalised C-alpha test was applied to the original data and then, in order to determine a permuted p-value, to repeated permutations of the case/control status and covariate data (see Text S1 for details of the standard permutation approach). We performed two separate analyses with and without adjustment for the lead MHC SNP for T1D, rs9268645. Assuming there are approximately 30,000 genes in the human genome [25], a p-value of less than 0.05/30,000 = 1.7×10^−6^ is required to ensure genome-wide significance. Hence for each analysis, we performed 600,000 permutations and declared genome-wide significance for a given gene if less than 1 of 600,000 (<1.7×10^−6^) permutations resulted in a C-alpha test statistic larger than the original.

## Results

### Simulation Study

The assumption that the C-alpha statistic is normally distributed under the null hypothesis depends on the quantity and independence of the variants considered as well as the accuracy of the individual estimates at each variant, which in turn depends on the sample size and the MAF. By considering regions of a fixed size and varying the minimum MAF of alleles considered and the sample size, we were able to effectively vary the number of variants and the allele frequency distribution in order to explore type I error and power.

#### Type I error

We began by considering evaluation of the type 1 error rate of the Generalised C-alpha test by performing simulations of 2,000 samples in a 50 kb region under a null model where there are no causal variants. Table 1 presents estimated type I errors of the Generalised C-alpha test applied to a quantitative trait and a binary trait (where the binary trait is a dichotomised version of the quantitative trait). Over all simulations, the mean number of rare variants with at least 4 copies of the minor allele (0.2%<MAF<1%) was 34; and with at least 10 copies (0.5%<MAF<1%) was 15. Results indicate that the type I error of the Generalised C-alpha tests applied to both the quantitative and the binary trait is well calibrated.

**Table 1:** Null simulations.

MinimumMAF %	Mean no. of variants in region	Type I error rates for significance level (95% Confidence Interval)			
		1×10^−5^	1×10^−4^	1×10^−3^	1×10^−2^
**Generalised C-alpha Test applied to a quantitative trait**					
0.2	34	<0.00001 (0.00000–0.00003)	0.00008 (0.00001–0.00016)	0.00081 (0.00059–0.00104)	0.00947 (0.00870–0.01025)
0.5	15	0.00001 (0.00000–0.00003)	0.00009 (0.00002–0.00016)	0.00102 (0.00079–0.00126)	0.00993 (0.00919–0.01067)
**Generalised C-alpha Test applied to a dichotomised version of a quantitative trait**					
0.2	34	<0.00001 (0.00000–0.00003)	0.00010 (0.00002–0.00018)	0.00091(0.00067–0.00115)	0.00952 (0.00875–0.01030)
0.5	15	0.00001 (0.00000–0.00003)	0.00013 (0.00005–0.00021)	0.00115(0.00090–0.00141)	0.01061 (0.00984–0.01137)

Observed type I errors at selected significance levels for the Generalised C-alpha test for association with a quantitative trait and a dichotomised version of a quantitative trait in a 50 kb region where the rare variants tested do not account for any of the trait variance. Tests only consider variants in the region with a maximum MAF of 1% and a minimum MAF as indicated in the table. Type I error is estimated over 100,000 replicates of data for a sample of size 2,000. Significance in each replicate of data is assessed empirically by random permutation of the quantitative trait value and recalculation of the test statistic 1,000 times as described in Text S1.

#### Power comparison

**Figure 1:**
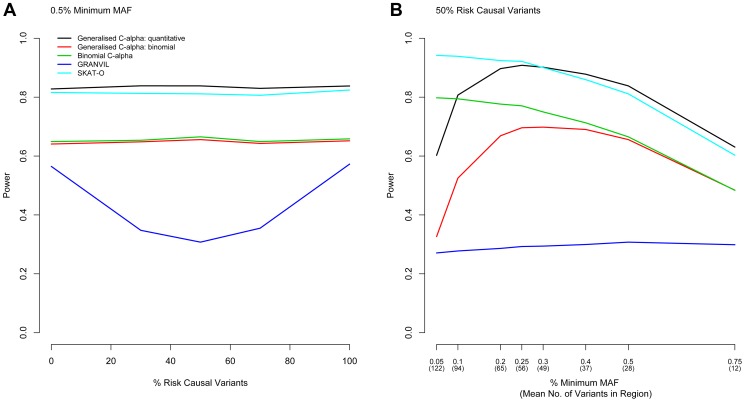
Power Comparisons. Power to detect association in a region is shown for the Generalised C-alpha test, SKAT-O and the GRANVIL test applied directly to the quantitative trait and for the Generalised C-alpha and the Binomial C-alpha tests applied to the dichotomised quantitative trait. (A) Power is shown as a function of the percentage of causal variants in a region of size 100 kb that are risk as opposed to protective when the minimum MAF of variants considered is fixed at 0.5% for a sample size of 10,000. Results show that as the proportion of risk causal variants approaches 50%, the C-alpha and SKAT-O tests maintain power and that the Generalised C-alpha applied directly to the quantitative trait has optimal power. (B) Power is also shown as a function of the minimum MAF of variants considered when the percentage of risk causal variants in a region of size 100 kb is fixed at 50% for a sample 10,000 individuals. Results show that the power of the Generalised C-alpha test is optimal for variants with MAF>∼0.3% but SKAT-O is optimal for lower MAF. For quantitative traits, the power of the Generalised C-alpha test remains better than the Binomial C-alpha applied to a dichotomized version of the trait as long as variants have MAF>∼0.1%. For binary traits, the Binomial C-alpha test has greater or equivalent power than the Generalised C-alpha test.

Next, we considered evaluation of the power of the Generalised C-alpha test by performing simulations of 5,000 and 10,000 samples in a 100 kb region under a range of trait association models. In all simulations, we assume that the maximum MAF of any causal variant is 1%, and the total MAF of causal variants within the gene is 5%, which together account for 0.6% of the trait variance. Simulation results evaluating power are shown in Figure 1 for 10,000 samples and in Figure S1 for 5,000 samples. The Generalised C-Alpha tests, the Binomial C-alpha and SKAT-O are robust to the presence of a mixture of risk and protective variants.

For quantitative traits and sufficiently large minimum MAF (see asymptotic properties), the Generalised C-alpha performed better than all the other tests compared. In the examples we selected, it performed equally as well or better than SKAT-O for variants with more than ∼15–25 copies of the minor allele (MAF>∼0.3% for 5,000 samples or MAF>∼0.25% for 10,000 samples) for any combination of risk or protective variants (only shown for 50% risk causal variants). However, the SKAT-O was optimal for variants with fewer copies of the minor allele. In our qualitative analyses of a binary trait, the Binomial C-alpha test and the Generalised C-alpha test were comparable for variants with MAF>∼0.5% but the power of the Generalised C-alpha test declined for variants with fewer than ∼15–20 copies of the minor allele (MAF<∼0.3% for 5,000 samples and MAF<∼0.2% for 10,000 samples).

#### Asymptotic properties

The power of the Generalised C-alpha test applied to the quantitative and the dichotomised traits decreases rapidly as the number of copies of the minor allele for included rare variants falls below ∼10 in the models we have considered (MAF<∼0.2% for 5,000 samples or MAF<∼0.1% for 10,000 samples). Rapid decreases in power with decreasing MAF are likely to be a consequence of increasing sparseness leading to violation of the assumptions of asymptotic normality in the Generalised C-alpha test. Of course, in a given region, the total number of variants considered increases as the minimum MAF decreases – in simulations for 10,000 individuals, the number of variants in our simulated 100 kb region when minimum MAF is 0.5% is 28 increasing to 94 for a minimum MAF of 0.1% - and losses in power are also a consequence of an increased number of non-causal variants being included in this total, but this factor affects the power of all the tests similarly (Figure S2).

#### Computation time

Computation time for the Generalised C-alpha depends on the sample size, the number of markers and the method used to estimate the normally distributed measures of effect at each variant. To analyse all ∼160 markers sequenced on 5,000 or 10,000 individuals in a 100 kb region and obtain permuted p-values with 1,000 permutations in a Generalised C-alpha test of association required ∼5.0 s and ∼10 s, respectively, for a quantitative trait (using estimates of effect derived from linear regression) and ∼20% longer for a binary trait (using estimates of effect derived from logistic regression). Increasing the number of permutations to 100,000 increased the run times ∼20-fold. Halving the number of markers analysed only marginally reduced run times. These estimates were based on simple code programmed in R and run on a Unix operating system. Coding in a language that allows faster numerical computation times is expected to reduce run times.

### Rare Variant Analysis of Imputed Data with T1D

After QC and imputation, the WTCCC data comprised 2,938 T1D cases and 1,963 controls with directly or imputed genotypes available at 490,888 SNPs with 0<MAF<1%, located in 1,611 distinct genes on chromosome 6; gene boundaries were identified from the UCSC human genome database (build 37). Table 2 shows the genes demonstrating genome-wide significant evidence of rare variant association with type-1 diabetes on chromosome 6. Genome-wide significant (Bonferroni correction for 30,000 genes at a 5% significance level: *p*<1.7×10^−6^) evidence of association with T1D were observed with rare variants in 17 genes throughout the 7.5 Mb extended Major Histocompatibility Complex (MHC) region (ranging from the GNL1 gene to the COL11A2 gene). The strongest signal of association was observed at C6orf10 (Z_NORM_ = 89.1, *p*<1.7×10^−6^), which contains rare variants previously implicated in susceptibility to T1D [26].

**Table 2:** Genes demonstrating genome-wide significant evidence of rare variant association with type-1 diabetes on chromosome 6.

Gene symbol	NCBI Build 37 chromosome 6 position (BP)		Number of rare variants	Unconditional analysis^a^	Conditional analysis: adjusted for lead MHC SNP^b^	
	Start	Stop		*Z_NORM_*	*Z_NORM_*	*p*
**Very rare variation 0.1%<MAF<0.5%**						
*HLA-DRB5*	32,485,162	32,557,562	189	60.5	40.3	5.2×10^−6^
**Rare variation 0.5%<MAF<1%**						
*GNL1*	30,513,695	30,525,008	9	51.0	14.1	1.7×10^−6^
*DHX16*	30,620,896	30,640,830	7	45.5	14.0	3.5×10^−6^
*C2*	31,865,561	31,913,448	12	20.7	12.9	4.0×10^−5^
*CFB*	31,895,265	31,919,860	8	19.9	9.7	1.3×10^−4^
*TNXB*	32,008,931	32,077,151	21	29.9	34.5	<1.7×10^−6^
*AK123889*	32,223,487	32,233,615	18	41.2	24.1	1.0×10^−4^
*C6orf10*	32,256,302	32,339,656	97	89.1	57.8	3.0×10^−6^
*BTNL2*	32,362,512	32,374,900	6	26.0	15.7	<1.7×10^−6^
*HLA-DRB5*	32,485,162	32,557,562	62	43.2	29.4	<1.7×10^−6^
*HLA-DRB6*	32,520,489	32,552,155	34	44.1	27.6	<2.5×10^−6^
*HLA-DQA2*	32,709,162	32,715,219	6	28.6	13.1	2.3×10^−5^
*HLA-DQB2*	32,723,875	32,731,330	6	18.5	17.0	<1.7×10^−6^
*TAP2*	32,781,499	32,806,547	18	21.8	18.9	<1.7×10^−6^
*HLA-DMB*	32,902,409	32,908,817	8	10.3	8.2	2.8×10^−5^
*BRD2*	32,936,436	32,949,281	13	14.9	11.2	8.7×10^−6^
*COL11A2*	33,130,468	33,160,245	13	31.2	17.4	1.7×10^−6^

a Genes with a permuted p-value less than 1.7×10^−6^ (indicating genome wide significance assuming a significance level of 5% and that there are 30,000 genes in the human genome [25]) in a Generalised C-alpha test.

b For these genes, results are also shown when effects are adjusted for the lead common MHC SNP (rs9268645). Both analyses are adjusted for 3 principal components to account for population structure. For the unconditional analysis results are based on 600,000 permutations; for the conditional analysis results are based on 575,000 permutations. MAF, minor allele frequency; BP, base pair; MAF: Minor Allele Frequency; MHC, Major histocompatibility complex; NCBI, National Center for Biotechnology Information.

Common SNPs in the MHC have been previously associated with T1D [17], [20]. Exactly which and how many loci in the MHC determine susceptibility remains unclear as a consequence of the high gene density and the strong association between alleles in the region. To take account of established associations in the MHC, we repeated our analyses on the genes with rare variants showing genome-wide significance evidence of association with T1D with adjustment for the lead MHC SNP (rs9268645) [17]. The common SNP explained the rare variant association in 11 of the MHC genes; 6 MHC genes achieved genome-wide significant evidence of rare variant association with T1D after adjustment for the lead MHC SNP.

## Discussion

We have developed the Generalised C-alpha test for the analysis of multiple rare variants that display a mixture of increaser and decreaser effects on a binary or quantitative trait. The Generalised C-alpha test is a score test combining routinely calculated Gaussian distributed measures of effect at multiple variants in order to increase the power to detect an effect at the gene, region or pathway level. The Binomial C-alpha test for binary traits, [14] and, more recently, SKAT-O [15], have been shown to have several advantages over previously proposed tests by Li and Leal [8], Madsen and Browning [9] and Price et al. [11]: most notably increased power in the presence of a mixture of increaser and decreaser effects. Our results confirm that the Generalised C-alpha test is also robust to the presence of bi-directional effects, with no apparent loss in power across a range of different mixtures.

The Generalised C-alpha test performs better than SKAT-O when the data is not too sparse: in our examples we showed the Generalised C-alpha was optimal as long as there were at least 15–25 copies of a minor allele at each rare variant. When data is sparse, so that either the sample size is too small and/or the MAF is too low, estimates of allelic effects at each SNP are not robust, and the asymptotic assumptions on which the Generalised C-alpha test are based are inappropriate. Similarly, for testing rare variant association with a binary trait, we have shown that the Generalised C-alpha test has lower power that the Binomial C-alpha test in the presence of variants with very low minor allele counts: a minimum MAF>∼0.5% is recommended in order to achieve comparable power in these tests.

In any application, the Generalised C-alpha test works on the assumptions that there are (i) a sufficiently large set of variants; (ii) that estimates of effect based on these variants are robust and independent and; (iii) normally distributed. These assumptions are often unrealistic: they are violated for example, in the presence of linkage disequilibrium, small sample size, low MAF or few variants. Hence, it is imperative that permutation testing is employed for accurate estimation of significance. For analysis of the whole genome, 1,000 permutations, for which a simply coded version of the test can be run in a matter of seconds, is recommended as a first approach; regions where the test is significant with a p-value<0.001 can then be rerun with 100,000 or more permutations for an accurate estimate of genome-wide significance.

Unlike the Binomial C-alpha test, the Generalised C-alpha test can naturally adjust for additional covariates and can easily incorporate imputed variation. Unlike SKAT-O, the Generalised C-alpha test can be applied to summary statistics, without requirement of the individual level genotype data. For example, the Generalised C-alpha test can be quickly and easily applied to published data. However, this is recommended only for discovery as permutation testing cannot be implemented in this case and test statistics are likely to be inflated leading to increased type I errors: In this case, any regions identified would require further investigation for any confirmation of association.

Evaluation of rare variants extracted from existing GWAS data via imputation up to re-sequencing reference panels, such as those made available by the 1000 Genomes Project, has been demonstrated to be feasible [18]. We applied the Generalised C-alpha test to rare variants imputed into the WTCCC T1D GWAS across the MHC where genes have been shown to play the single most important role in susceptibility to T1D in both common variant and haplotype analyses. Genome-wide significant association with T1D, independent of the lead common variant GWAS signal in the region, was observed at multiple genes. These included HLA class II genes, DR and DQ, where coding polymorphisms have been shown to account for most of the association with T1D observed at the HLA locus [27], [28], [29]. The identification of rare disease-associated variants within genes in this region highlights the complex genetic architecture of T1D in the MHC, and requires further investigation to disentangle the effects of common and rare variation on immune disease susceptibility.

In summary, the Generalised C-alpha test is a novel, flexible and powerful method for the analysis of rare genetic variation. There is no single alternative test, amongst those we have considered, that is uniformly most powerful over all models and genetic architectures. Our test, however, has the unique advantage that it can be applied to summary statistics from published literature, without the need for individual level genetic data. The fact that the Generalised C-alpha test simply aggregates data from summary statistics allows for great flexibility in general allowing direct application to both binary and quantitative traits, to population (using summary statistics from generalized linear models, as illustrated here) and family based data (using summary statistics from the transmission disequilibrium test, for example), and to imputed genotype data whilst simultaneously allowing for the adjustment of additional covariates. We are already using the method in our analyses and it is currently implemented using the R-PLINK/SEQ library available from: http://atgu.mgh.harvard.edu/plinkseq/. R package is available from http://www.well.ox.ac.uk/~rivas/calphanorm.tar.gz.

## Supporting Information

Figure S1 **Power Comparisons**. Power to detect association in a region is shown for the Generalised C-alpha test, SKAT-O and the GRANVIL test applied directly to the quantitative trait and for the Generalised C-alpha and the Binomial C-alpha tests applied to the dichotomised quantitative trait. (A) Power is shown as a function of the percentage of causal variants in a region of size 100 kb that are risk as opposed to protective when the minimum MAF of variants considered is fixed at 0.5% for a sample size of 5,000. Results show that as the proportion of risk causal variants approaches 50%, the C-alpha and SKAT-O tests maintain power and that the Generalised C-alpha applied directly to the quantitative trait has optimal power. (B) Power is also shown as a function of the minimum MAF of variants considered when the percentage of risk causal variants in a region of size 100 kb is fixed at 50% for a sample 10,000 individuals. Results show that the power of the Generalised C-alpha test is optimal for variants with MAF>∼0.3% but the SKAT-O is optimal for lower MAF. For quantitative traits, the power of the Generalised C-alpha test remains better than the Binomial C-alpha applied to a dichotomized version of the trait as long as variants have MAF>∼0.12%. For binary traits, the Binomial C-alpha test has greater or equivalent power than the Generalised C-alpha test.
(TIF)

Figure S2 **Power By Region Size.** Power is shown as a function of region size when the percentage of risk causal variants is fixed at 50%, the minimum MAF of variants considered is fixed at 0.5% for a sample size of 10,000 individuals. Here, the region size is a proxy for the number of variants considered and results show that power decreases for all methods as the number of non-causal variants included increases. Results are presented for a model assuming a total MAF of 5% for all causal variants in the region, a maximum MAF of any individual causal variant of 1% and where causal variants account for 0.6% of the phenotypic variance. The trait mean is determined by the net effect of the risk causal variants, which serve to increase the mean trait value, and the protective causal variants, which serve to decrease the trait mean. Power is estimated at a 5% significance level over 10,000 replicates of data. Significance in each replicate of data is assessed empirically by random permutation of the trait value and recalculation of the test statistic: permutation occurs 1000 times to ensure accurate assessment at a significance level of 5%.
(TIF)

Text S1 A flexible approach for the analysis of rare variants allowing for a mixture of effects on binary or quantitative traits: Supplementary Methods
(DOCX)
