# Supplementary figures and images for: A Flexible Approach for the Analysis of Rare Variants Allowing for a Mixture of Effects on Binary or Quantitative Traits
Source: PLoS Genet. 2013 Aug 15;9(8):e1003694. doi: 10.1371/journal.pgen.1003694 (PMC3744430; doi:10.1371/journal.pgen.1003694)

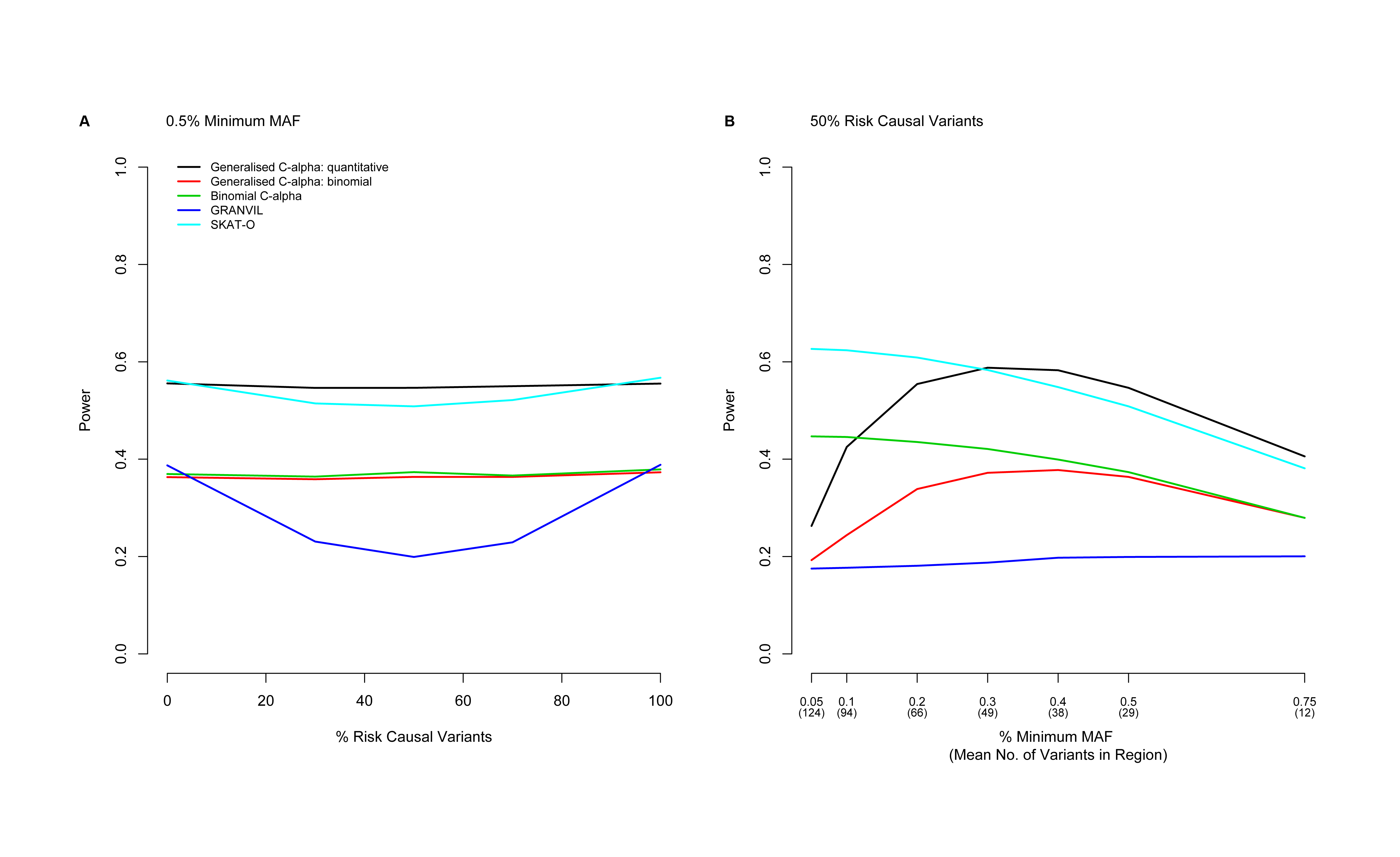

Supplement: Figure S1 — Power Comparisons. Power to detect association in a region is shown for the Generalised C-alpha test, SKAT-O and the GRANVIL test applied directly to the quantitative trait and for the Generalised C-alpha and the Binomial C-alpha tests applied to the dichotomised quantitative trait. (A) Power is shown as a function of the percentage of causal variants in a region of size 100 kb that are risk as opposed to protective when the minimum MAF of variants considered is fixed at 0.5% for a sample size of 5,000. Results show that as the proportion of risk causal variants approaches 50%, the C-alpha and SKAT-O tests maintain power and that the Generalised C-alpha applied directly to the quantitative trait has optimal power. (B) Power is also shown as a function of the minimum MAF of variants considered when the percentage of risk causal variants in a region of size 100 kb is fixed at 50% for a sample 10,000 individuals. Results show that the power of the Generalised C-alpha test is optimal for variants with MAF>∼0.3% but the SKAT-O is optimal for lower MAF. For quantitative traits, the power of the Generalised C-alpha test remains better than the Binomial C-alpha applied to a dichotomized version of the trait as long as variants have MAF>∼0.12%. For binary traits, the Binomial C-alpha test has greater or equivalent power than the Generalised C-alpha test. (TIF) [file pgen.1003694.s001.tif]

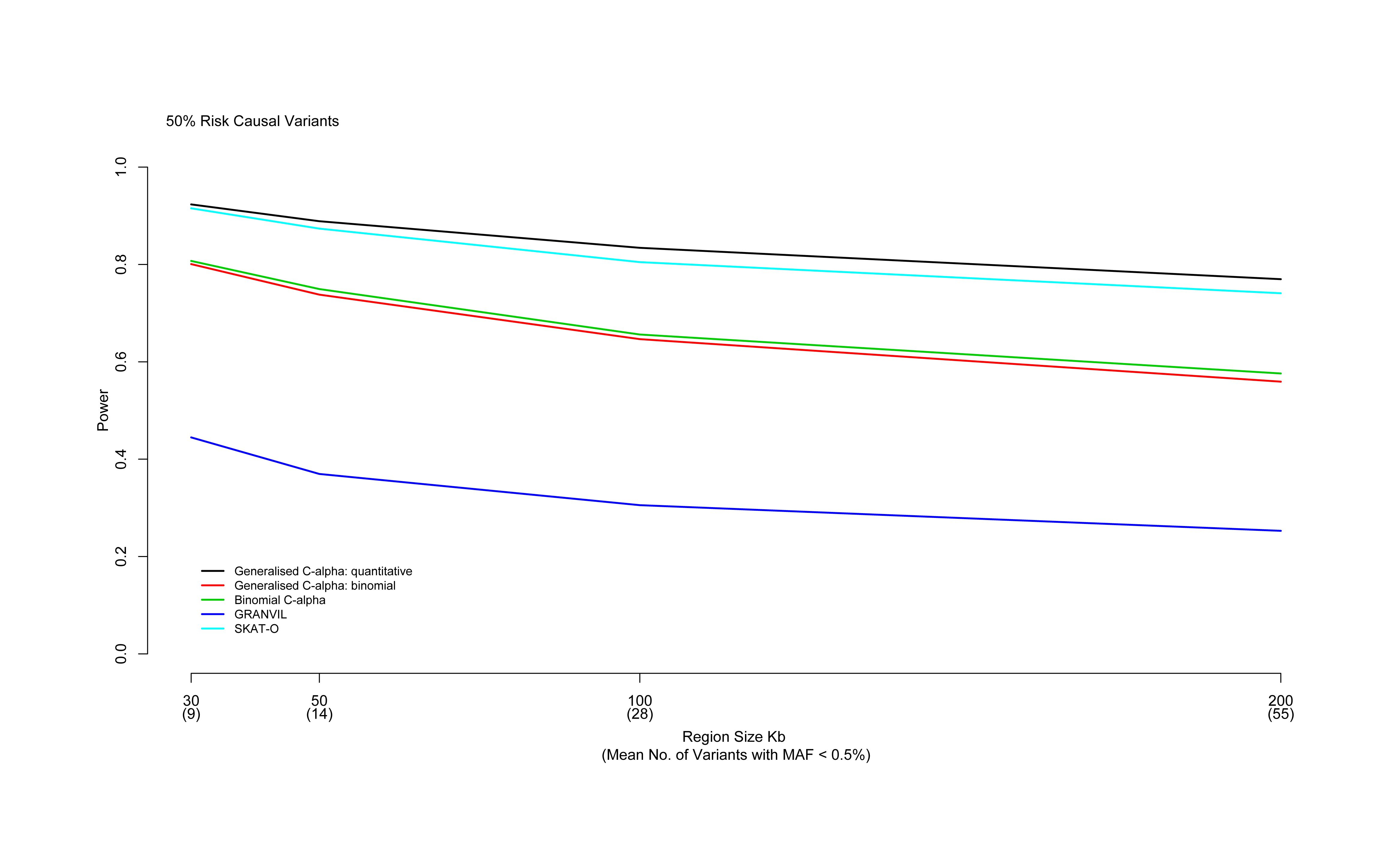

Supplement: Figure S2 — Power By Region Size. Power is shown as a function of region size when the percentage of risk causal variants is fixed at 50%, the minimum MAF of variants considered is fixed at 0.5% for a sample size of 10,000 individuals. Here, the region size is a proxy for the number of variants considered and results show that power decreases for all methods as the number of non-causal variants included increases. Results are presented for a model assuming a total MAF of 5% for all causal variants in the region, a maximum MAF of any individual causal variant of 1% and where causal variants account for 0.6% of the phenotypic variance. The trait mean is determined by the net effect of the risk causal variants, which serve to increase the mean trait value, and the protective causal variants, which serve to decrease the trait mean. Power is estimated at a 5% significance level over 10,000 replicates of data. Significance in each replicate of data is assessed empirically by random permutation of the trait value and recalculation of the test statistic: permutation occurs 1000 times to ensure accurate assessment at a significance level of 5%. (TIF) [file pgen.1003694.s002.tif]
