# Supplementary material for: A Flexible Approach for the Analysis of Rare Variants Allowing for a Mixture of Effects on Binary or Quantitative Traits
Source: PLoS Genet. 2013 Aug 15;9(8):e1003694. doi: 10.1371/journal.pgen.1003694 (PMC3744430; doi:10.1371/journal.pgen.1003694)
Supplement: Text S1 — A flexible approach for the analysis of rare variants allowing for a mixture of effects on binary or quantitative traits: Supplementary Methods (DOCX) [file pgen.1003694.s003.docx]

**A flexible approach for the analysis of rare variants allowing for a mixture of effects on binary or quantitative traits:**

**Supplementary Methods**

***Simulations***

We have performed simulations to evaluate the performance of the Generalised C-alpha test for the identification of rare variant associations with a quantitative or a qualitative trait. We considered a model for association of the trait with multiple causal variants in the same region, under the assumption that the mean trait value is determined by the net effect of risk and protective causal variants at which a minor allele is present. The trait association model was parameterised in terms of: (i) the maximum MAF, δ, of any individual causal variant; (ii) the total MAF, *Q*, of all causal variants (iii) the proportion of risk, γ (0 ≤ γ ≤ 1), as opposed to protective, causal variants and (iii) their joint contribution to the phenotypic variance, expressed as 100λ%. For each model, we generated a single replicate of data as follows:

1. Generate an ancestral recombination graph [[1](#_ENREF_1)] for a population of 2N haplotypes from a realisation of the coalescent process with recombination, obtained using the MS software [[2](#_ENREF_2)]. We assumed a mutation rate of 10^-8^􏰃per base (in each generation) and a recombination rate of 1 cM per Mb, for an effective population size of 10,000 individuals corresponding to scaled recombination and mutation rates of *ρ* = *θ* = 20 across the 50kb region [[3](#_ENREF_3)].
2. Calculate the MAF at each variant across the population, denoted *q*_j_ for the *j*th locus. Select a random subset of variants as causal, each with MAF *q*_j_ < δ, and with total MAF of approximately *Q*. Randomly assign 100γ% of these causal variants to be risk and the remainder as protective.
3. Pair the 2N chromosomes at random to form a cohort of N individuals to be genotyped for association testing For the *i*th individual, determine the number of minor alleles across all risk causal variants, denoted *m^r^_i_*. and across all protective causal variants, denoted *m^p^_i_*. Under the assumption that the trait mean is determined by the net effect of a combination of risk and protective causal variants, we simulate the phenotypic value, *y_i_*, from a N(*m^r^_i_* – *m^p^_i_*, σ) distribution. The standard deviation, σ, is determined by the spectrum of causal variants and their joint contribution, λ, to the phenotypic variance.
4. Record the genotypes of each individual in the cohort at all variants with a MAF less than 1% in the cohort. Apply the proposed tests and record the P-values.

For each model, we created 10,000 replicates of data and recorded the proportion of replicates in which the P-value of the proposed test was less than a given significance threshold.

***Standard Permutation Approach***

Standard permutation testing proceeds by random reassignment, or permutation, of the combined phenotypic and covariate data across all individuals. Effectively, each individual in the study is randomly reassigned the phenotypic and covariate data belonging to another individual in the study for each permutation of the data. In this manner, the correlation structure between phenotype and covariate data is maintained and the genetic effect adjusting for the covariates is properly tested. For this permuted data, estimates at each variant are recalculated and a permuted C-alpha test statistic derived.. This process is repeated *P* times to obtain *P* permuted C-alpha test statistic values. The permuted p-value is then the proportion of the *P* permuted C-alpha test statistics that are greater than the original C-alpha test statistic, that is, the C-alpha statistic calculated from the original data. Clearly, genotype and covariate data are required for standard permutation testing. For the simulations, our standard permutation approach is implemented for each replicate of the data.

**References**

1. Griffiths RD, Marjoram P (1997) An ancestral recombination graph. In: Donnelly P, Tavare S, editors. Progress in Population Genetics. New York: Springer 257-270.

2. Hudson RR (2002) Generating samples under a Wright-Fisher neutral model of genetic variation. Bioinformatics 18: 337-338.

3. Nordberg M (2001) Coalescent theory. In: Balding DJ, Bishop M, Cannings C, editors. Handbook of Statistical Genetics. Chichester: Wiley 179-212.
